# Opportunities and difficulties with providing child healthcare through team-based visits in child healthcare services – a focus group study

**DOI:** 10.1186/s12875-025-02981-0

**Published:** 2025-09-08

**Authors:** Ulrika Svea Nygren, Leif Eriksson, Håkan Sandberg, Ylva Tindberg, Lena Nordgren

**Affiliations:** 1 Department of Public Health and Caring Sciences, Uppsala University, BMC, Box 564, Uppsala, 751 22 Sweden; 2 Center for Clinical Research Sörmland, Uppsala University, Box 529, Eskilstuna, 631 07 Sweden; 3 School of Health, Care and Social Welfare, Mälardalen University, Box 883, Västerås, 721 23 Sweden; 4 Department of Women’s and Children’s Health, Uppsala University, Akademiska sjukhuset, Uppsala, 751 85 Sweden

**Keywords:** Child healthcare services, Focus group discussion, Interprofessional teamwork, Team-based visits, Team competence

## Abstract

**Background:**

The Swedish Child Healthcare services (CHS) includes team-based visits. Team-based visits are defined as physical meetings where different professionals, including the child and his or her parents, participate at the same time at the same place. This study aimed to explore healthcare professionals’ experiences of these visits to better understand the opportunities and difficulties in meeting the unique needs of children and their families through team-based visits.

**Methods:**

An explorative qualitative design. Data were obtained through seven digital focus group interviews (FGDs) with 34 professionals within CHS, following the guidelines provided by Krueger and Casey.

**Results:**

All professional groups perceived great opportunities with team-based visits. Optimal team-based visits meant accessible and safe family support, based on children’s and families’ needs. Trustful relationships with someone in the team, can promote families’ trust in the team, the assessment process, and healthcare organizations. For optimal team-based visits in CHS, professional competence, as well as organizational structures, are required. However, without enabling factors, here can be opposite effects on all those involved.

**Conclusions:**

Optimal team-based visits in CHS delivered in the right way, at the right time, by the right individuals can be an efficient and comprehensive way to meet the unique needs of children and families.

**Supplementary Information:**

The online version contains supplementary material available at 10.1186/s12875-025-02981-0.

## Introduction

The Convention on the Rights of the Child, which became Swedish law (2018:1197) on January 1, 2020, declares all children’s right to have the highest attainable standard of health and access to Child Healthcare Services (CHS) [1]. Globally, it is recommended that CHS be delivered by interprofessional teams within a framework of proportionate universalism, aiming to reduce health inequalities among children [2–4]. According to Carey (2015), proportionate universalism implies having CHS *“with a scale and intensity that are proportionate to the level of disadvantage*” (p. 1, row 9) [4]. However, there are challenges regarding how to apply CHS proportionally, as well as how to identify the prerequisites and needs [2, 4]. Without this knowledge, there is a risk of continued disparities in both child health access to healthcare services, including coverage of CHS. Such inequities in child health could negatively affect the welfare of society for decades [5–7].

The Swedish National Board of Health and Welfare’s instructions for CHS, together with a web-based national guide, constitute the Swedish National Child Healthcare Program (NCHP) [8, 9]. The NCHP is a three-tier program, comprising a combination of universal, selective, and targeted activities for all children between 0 and 6 years of age [8–10]. The program describes interprofessional teamwork among nurses, physicians, and psychologists [8, 9, 11]. Nurses in CHS should have a specialist education either as public health nurses or pediatric nurses, and physicians in CHS should be specialized in general medicine, pediatrics or child psychiatry [8, 9]. The CHS nurses are responsible for the ongoing CHS, and the physicians are the nurses’ main collaborative partners [8, 11]. Psychologists work directly and indirectly with children and parents and support professionals in CHS. Psychologists and other professionals usually meet the child and his or her family following a referral from a nurse or a physician [8, 9]. The universal basis of the NCHP comprises 14 exploratory, promotional, and preventive health visits to the nurse at specific ages. The first health visit with the nurse is recommended to take place in the child’s home, followed by health visits at Child Healthcare Centers (CHCs) or Family Centers (FCs), and another health visit in children’s home at 8 months. In addition, the Swedish NCHP includes universal team-based visits, including a nurse and a physician, are offered at 4 weeks, 6 months, 12 months, and 2.5–3 years, and take place at CHCs or FCs. Also targeted team-based visits are recommended for children with extensive needs and can also be conducted in other places, e.g., the child’s home [8, 9]. To our knowledge, there are no studies exploring team-based visits in CHS, where different professionals participate at the same health visit, as described in the Swedish NCHP. Internationally, studies relating to team-based care in CHS describe the provision of CHS divided between physicians and nurses [12–14]. Overall, team-based care is described as a shared care model providing health care to individuals, or families, by at least two healthcare providers to accomplish shared goals and to meet patients’ need [15, 16]. However, we did find studies where patients with diseases or illness participated in team meetings [17–19]. In the CHS context, there are studies describing targeted team-based home visits where nurses collaborate with dentists or dental nurses or social workers in joint home visits for vulnerable groups [20–22]. Prior studies in the Swedish CHS show that healthcare professionals require team-based visits to be delivered by interprofessional teams in line with proportionate universalism [10]. However, there were hindering factors for team-based visits, stemming from organizational structures and lack of human resources [10]. An understanding of factors influencing teams within CHS is crucial for implementing interprofessional collaboration in practice [2, 23, 24]. Factors associated with teamwork are connected to the individual, the team, and the organization [24–26].

Previous research describes the importance of understanding the underlying mechanisms in well-functioning teams, as competences, structures, and processes within the team, and the context influencing the teams’ ability to deliver complex clinical tasks and quality of care [25–28]. Being aware of the challenges faced by healthcare professionals who interact with families is important when designing teams that can meet the needs of children and families in CHS [26]. To fully understand factors that facilitate and hinder team-based visits in CHS, as well as know how to organize optimal team-based visits, it is important to study the healthcare professionals’ perspective [2]. This knowledge can be used to achieve an even and equitable distribution of team-based visits in CHS and contribute to equality and equity in children’s health [2]. The universal and targeted team-based visits in CHS and is considered important to meet the unique needs of the child and his or her family [8, 9, 29]. Team-based visits are defined as physical meetings where different professionals, including the child and his or her parents, participate at the same time and at the same place [8, 9, 29]. Without relevant knowledge of teams, they exist in name only [27]. This study is part of a larger project focused on interprofessional teamwork within the Swedish CHS. The present study aimed to explore the experiences of team-based visits among nurses, physicians, and psychologists in Swedish CHS to better understand the opportunities and difficulties in meeting the unique needs of children and their families through such visits.

## Methods

### Study design

This study used a qualitative exploratory research design [30]. Consolidated criteria for reporting qualitative research (i.e., COREQ guidelines) were followed when reporting the study (see S1 Checklist) [31]. Data were obtained through focus group discussions (FGDs), following the guidelines provided by Krueger and Casey [30]. Focus group discussions are suitable when facing complex themes. In this study, they were used to gain an in-depth understanding of the phenomenon of team-based visits in order to effectively illuminate different aspects [30]. Interactions between participants in FGDs can highlight the similarities and differences in participants’ opinions about team-based visits and provide a broad range of perspectives and experiences of such visits in CHS [30, 31].

### Participants and setting

The setting for this study was the Swedish CHS. The participants (*n* = 34) included healthcare professionals in CHS from seven out of the 21 healthcare regions in Sweden. To obtain in-depth knowledge about team-based visits, purposeful sampling was used by selecting individuals who were especially knowledgeable about or experienced with this practice [30]. To gain a broad perspective, nurses, physicians, and psychologists from different settings and different parts of Sweden were included. Some participants in the FGDs worked within the same healthcare region and knew each other. The healthcare regions represented different geographical areas from north to south, including big cities and sparsely populated areas, as well as differences in socioeconomic conditions. Although the health of children in Sweden is ranked among the best in the world, there are increasing differences in health outcomes. Sweden has a public tax-financed healthcare system, which provides CHS to all children. The Swedish state tries to minimize differences through healthcare resource allocation based on the socioeconomic care need index. The first author contacted five regional Main Child Healthcare Units (MCHUs) across Sweden, including care developers and medical directors who had participated in a national effort to implement team-based visits. The first author works as a care developer and recruited healthcare professionals via e-mail. Moreover, some participants were recruited through a national lecture within a professional education program for CHS providers. Information about the study was sent to current MCHUs, who then forwarded it to potential participants. Initially, there were 40 healthcare professionals who were interested in participating. However, we over-recruited potential participants considering that participants might not always be available on the occasions. Three of the interested nurses and three physicians could not participate because the scheduled FGDs were not at a suitable time. The MCHUs cited lack of time associated with the Covid-19 pandemic and lack of experience with team-based visits as obstacles for registered interest. In total, seven FGDs with representations from seven healthcare regions were conducted with nurses (*n* = 10), physicians (*n* = 13), and psychologists (*n* = 11) engaged in CHS. Some of the participants knew the first author because they have worked in the same region or had met in national contexts. Table 1 provides the characteristics of the participants in the FGDs.

**Table 1:** Overview of the participants in the focus groups

Group	Profession	Number of participants	Gender	Years in CHS	Time in CHS (%)	Workplace	
1	Mixed	4	Female	4–25	50–100	CHC, FC, MCHU	
2	Psychologists	3	Female	2–9	50–100	CHC, PSYCH Clinic	
3	Psychologists	7	Female	1–25	50–100	CHC, FC, PSYCH Clinic	
4	Physicians	5	Female	1,5–49	5–65	CHC, FC, PED Clinic, MCHU	
5	Physicians	7	Female, Male	1–30	5–38	CHC, FC, MCHU	
6	Nurses	5	Female	2,5–35	90–100	CHC FC	
7	Nurses	3	Female	5–10	100	CHC^,^ FC	

*CHC* Child Healthcare Center, *FC* Family Center, *MCHU* Main Child Healthcare Units, *PSYCH Clinic* Psychologist Clinic, *PED Clinic* Pediatric Clinic

Child healthcare services are provided at CHCs or FCs, either in separate facilities or in close connection to a healthcare center. In FCs, the CHS is co-located with other child- and parental services [8]. Certain aspects of CHS can be conducted in other locations, i.e., psychologist clinics, MCHU, healthcare centers, and specialist CHS at pediatric clinics. The study participants worked at the above-mentioned workplaces (See Table 1). All healthcare professionals operate under the supervision of the National Board of Health and Welfare [8]. The CHS describes the standards for competence for nurses, physicians, and psychologists, including the responsibilities for each profession. These standards are available at the web-based national guide [9]. For nurses and physicians, there is a need for specialist education in Pediatrics or Public Health. Psychologists must be licensed and have knowledge about consultation, child development, and parenting [9].

### Data collection and procedure

A semi-structured interview guide was developed and pilot tested in March 2021 (Table 2). The pilot FGD included two nurses, one physician, and one psychologist. The rationale for having a mixed pilot FGD was to test the questions with professionals involved. Since only minor adjustments were made in the interview guide and the pilot group generated rich data in line with the aim of the study, the pilot group was included in the study. The interview guide constituted a framework for the discussions. Probes such as ‘can you describe more (…)’ and ‘what do you mean? (…)’ were used to gain a deeper understanding of the participants’ descriptions. Six homogenous FGDs, based on the profession, were held between February 2022 and March 2022, with 3–7 participants in each FGD. The first author (USN) moderated all the FGDs, while the last author (LN) acted as an assistant moderator. The moderator (USN) is a public health nurse with experience from CHS and in leading groups, care developer and Doctor of Medicine. The assistant moderator has experience working with teams in other healthcare contexts and conducting FGDs. Each FGD lasted approximately 75 min. The discussions took place digitally via Microsoft Teams 1.5 and were then recorded and transcribed verbatim by the first author. By using the FGD methodology, we sought to stimulate participants to share their views, respond to each other’s opinions [30], and raise new topics. Participants were encouraged to respond to each other’s opinions. In almost all group discussions, participants engaged in the conversations and responded to each other’s opinions and supplementary questions equally. However, the level of interactions among participants varied. In the two groups with seven participants, one psychologist and one physician made only a few comments. The group members in the smaller FGDs (3–5 participants) responded to each other to a greater extent than in the larger groups. There was a good conversational atmosphere, with a lot of recognition and humor among the participants. Most of the participants showed great commitment to CHS and acknowledged its importance for children’s health and development. However, sometimes they were surprised regarding their different circumstances and experiences of the team-based visits described. Field notes were made during the FGDs and at the end of the FGDs, the assistant moderator summarized the discussions and gave the participants an opportunity to comment or remark on the summary.

**Table 2:** Interview guide for the focus groups

Introduction		
• What are your first thoughts about team-based visits in Child Healthcare Service (CHS)? • Are there any differences between teamwork and team-based visits within CHS and teamwork and team-based visits in other healthcare services?		
Key questions		
• What opportunities have you experienced in your work to meet the unique needs of the children and their families through team-based visits in CHS? • What are the facilitating factors? • When do we need team-based visits?		
• What difficulties have you experienced in your work in meeting the unique needs of the children and their families through team-based visits in CHS? • What are the obstacles? • When do we not need team-based visits?		
• If you were allowed to wish freely, what is an optimal team-based visits? • Tell me about your experiences		
Conclusion and summary		
• Have we missed asking about something of significance according to team-based visits in CHS? • Do you want to add or clarify something before we end?		

### Data analysis

The data from the FGDs were systematically analyzed following an analysis guide developed by Krueger and Casey [30]. Their [31] method is suitable for analyzing such data, where the research questions can provide the initial framework for manifest analysis. In the second phase, the analysis becomes more latent [30]. Krueger and Casey recommend taking a step back after systematic analysis to compare the different categories and identify themes. This process means moving from the manifest to the latent content. It involves assessing the variability, passion, or intensity of the comments [30]. We conducted a transcript-based analysis with support from NVIVO version 1.6.1. These steps were followed: (1) The analysis process began during the FGDs, probing for detailed experiences and emotions, as well as a brief summary; (2) After each FGD, the first author and the last author reflected together to achieve a shared understanding of the content of the discussion; (3) The first author reviewed the transcripts from the digital focus groups and corrected any inaccuracies. Subsequently, she read the transcripts several times to grasp the overall sense; (4) The transcripts were then transferred in their entirety to NVivo, where meaningful units were coded with descriptive names, and codes relevant to the study’s aim were formulated and sorted under the headings *“Opportunities with team-based visits”* and *“Difficulties with team-based visits”*; (5) The analysis of the codes resulted in various categories and sub-categories; (6) All sub-categories under each category were sorted according to Nelson and Batalden (2007) and Drinkas’ (2016) theory of factors at different levels affecting healthcare teams [24, 26]: Child and family as well as Professionals and Organizations; (7) Considering the whole dataset and moving from the manifest to the latent content, an overall theme emerged related to opportunities and difficulties with team-based visits (Table 3). Verbatim quotations were used to illustrate the results, ensure validity, and verify the categorizations [30] **(**Table 3**).** The first (USN), second (LE), and last (LN) authors took part in the analysis. This involved repeated readings and reflections during the entire analytic process.

**Table 3:** Theme, categories and sub-categories theme

Optimal team-based visits were considered as resulting in synergy effects for all parties involved, while dysfunctional teams could have the opposite effect.		
	**Opportunities with team-based visits**	**Difficulties with team-based visits**
Categories	Child in the center	
Sub-categories		
Child and Family	Meeting the unique needs of the children and their families’, timely and accurate The child’s perspective becomes visible, and complex needs can be addressed	Certain issues of a sensitive character or uncomplicated matters are not suitable for team-based visits Lack of child perspective leads to inequitable and unequal child and family support
Professionals	Job satisfaction and sense of meaningful context	Fragmented, parallel, and sequential approach
Organizations	Child in the center should be the guiding principle for organization of team-based visits Colocation facilitates team-based visits	When management and professional perspectives take precedence over child’s perspective
Categories	Shared responsibilities and common goals	
Sub-categories		
Child and Family	Involvement and communication on equal terms	Quiet and shy children are a challenge
Professionals	Clarity regarding roles and goals through communication and shared responsibility for the goal fulfillment	Different views on goals and roles lead to confusion and missed goals
Organizations	Prioritizing based on national guidelines leads to equalities and equities in CHS	Competing interests lead to inequalities and inequities in CHS
Categories	Supportive approach and continuous trustful relations	
Sub-categories		
Child and Family	Strengthens families’ trust, confidence, and empowerment.	Misunderstandings, mistrust, and lack of confidence
Professionals	Flexibility and openness Work-related well-being and trust in each other	Paternalistic or educational approaches Mistrustful work-related relations
Organizations	Scheduled time for continuity in care and trustful relations throughout the whole organization	Mistrust within the healthcare organization
Categories	Competence and interprofessional learning	
Sub-categories		
Child and Family	Comprehensive, high quality, and safe support through a holistic view of the child in his or her family	Insufficient support in terms of quality and quantity
Professionals	Core team and extended team Problem-solving through mutual exchange Expertise through profession specific-, team- and child health care competences	Frustration and additional work Lack of qualified professionals
Organizations	Provision of the right human resources Effective use of competence in the organization	Ineffective use of professional competence

## Results

This section describes the opportunities and difficulties related to team-based visits in the CHS-context. All professional groups perceived great opportunities with team-based visits, as they could meet the unique needs of children and families, provided that the enabling prerequisites, structures, and processes existed within the team. The different conditions and resources of children and families, along with the attitudes and competences of team members, as well as the structures and processes within the team are all factors that influence the team’s ability to meet the needs of children and families. Additionally, organizational conditions and the broader context play a role. Optimal team-based visits were considered as resulting in synergy effects for all parties involved.

*You often get more than twice as far as you do alone. Especially with these slightly more complicated cases.* (Physician, Group 5)

However, participants described that the positive effects could be undermined if teams lacked certain individual and organizational conditions. Team-based visits could even have the opposite effect on children and their families, team members, and healthcare organizations when there were difficulties within the team. The effects of team-based visits, as well as the structures and processes important for the team’s ability to perform high quality team-based visits, are summarized under the categories that emerged.

### Child in the center

Participants considered the type of issue and the unique needs of children and families as being decisive for team-based visits. These visits, organized according to the family’s needs, were requested to prevent illness and promote health through early detection of specific needs.

*Well-functioning team-based visits… are the absolute best for the families and the child…* (Nurse, Group 7*)*.

Complex matters, such as risk assessments to determine the likelihood of neglect or maltreatment, were deemed appropriate for team-based visits. These visits could save time, alleviate concerns about the child and the family, and contribute to more accurate CHS assessments.

*… when there is something that is a little*,* maybe questionable… wondering how to handle it in the best way… how extremely valuable it is to have been two of you during the same visit*,* which the rest of you have said… the ability to discuss with each other.* (Physician, Group 5)

The child’s perspective is promoted by well-functioning team-based visits. A shared child- and family centered approach was perceived as important for ensuring that the child’s perspective is acknowledged and for professionals to feel they are effectively meeting the needs of children and families. Compared to general practitioners, pediatricians were considered more likely to understand from a child’s perspective. By participating in team-based visits, the psychologists and physicians felt closer to the families, and that they were part of a larger, meaningful context.

*In team-based visits*,* … We are here for the child*,* and we gather around the child*,* and we need to find a way to understand the child’s situation and agree on a common picture.* (Psychologist, Group 2)

The unique needs of children and families and how they are equipped should be the guiding principle for organizing team-based visits. All professional groups stressed the importance of a supportive organization to enable physical meetings, including having adequate human resources, locations, and rooms customized for team-based visits. Proximity to other professions, as seen in FCs, was considered a facilitating factor for optimal team-based visits.

*Because we have the midwives in the same corridor*,* we have had some team-based visits with the midwives during the pregnancy… at the end of the pregnancy…. it could be a concern or a family situation.* (Nurse, Group 7)

Some sensitive issues, such as conversations about mental illness or profession-specific uncomplicated conditions, were considered unsuitable for team-based visits. Difficulties arose from unequal access to team-based visits due to different organizational conditions. Several participants shared time between healthcare centers, pediatric clinics, or psychologist clinics, which could complicate scheduling and team affiliation. Organizational structures that prioritize management or professional perspectives over the child’s perspective can lead to fragmented, parallel, and sequential delivery of care.

### Shared responsibilities and common goals

Optimal teams collectively define and solve complex problems through mutual exchange. Participants requested clarity in content and purpose for the team-based visits, as well as clarification of the professionals’ roles. The family was viewed as being part of the team and could be involved through inclusive and open communication. Equal involvement of both parents was considered important.

*And equally… it is important to always ask both parents if there is something they want to discuss or bring up. There must be time for that, so that it’s not just our agenda.* (Physician, Group 4)

Communication about goals, norms, and shared values was described as important for consensus in team-based visits and throughout the whole organization. It was suggested that teams meet before and after visits to discuss goals and expectations, fostering meaningful interactions.

*… when I get to collaborate with a physician who sort of thinks in the same way as me about these children and dares to deal with the complex issues and the entire situation as well … it’s like a kind of security that everyone sees roughly the same thing….* (Psychologist, Group 3)

Shared responsibility within the team, together with the individual responsibility, was deemed important in team-based visits. The intention should be to complement each other on equal terms rather than compete with one another.

*… like what we have here and that everyone really thinks that everyone is needed in a different way. Really! I think that you see your piece in the puzzle, but that you understand that you need other people.* (Psychologist, Group 2)

Participants stated that responsibility and leadership within the team could vary depending on the reason for the team-based visit.

*Sometimes I took a step back and he took the lead and sometimes I took the lead, and he stepped back… and we listened in… so, when it works like this, it is fantastic. Then it’s fun too; it’s a colleague.* (Nurse, Group 6)

Professionals required methodological support and evidence to optimize team-based visits.

However, dealing with quiet and shy children could be a challenge in team-based visits, requiring flexibility within the teams. Furthermore, competing interests and different views on goals and roles within the team were viewed as problematic, and could lead to insecurity and confusion among families, as well as disharmony and inequality within teams. Organizational management with competing goals could lead to a lack of equality and equity in CHS.

### Supportive approach and continuous trustful relations

Optimal team-based visits could increase families’ confidence in their own abilities, namely instill trust and confidence. Establishing continuous, trustful relationships with someone in the team, usually a nurse, and being prepared for visits can promote families’ trust in the team, the assessment process, and healthcare organizations. Physicians viewed the team-based visits in CHS as unique compared to other team-based visits in healthcare services. Nurses’ role as key stakeholders and her/his continuous contact with the family were described as supporting the team’s goals.

*We are managing a capital of trust that we get with the CHS-nurse who already knows the family, so that we kind of ride that wave…so…it’s really much easier for me to come in and ask sensitive questions from the beginning because for me all the groundwork is done.* (Physician, Group 5)

Willingness to collaborate and positive attitudes among team members, such as a high degree of openness, respectfulness, responsibility, interest in the children and their families, were viewed as helping to build trust within the team and with families.

*It is very important to be available*flexible and open.(Physician, group 5)

All professional groups described various benefits, such as work-related well-being, job satisfaction, equality, security, reduced stress, and joy as outcomes of well-functioning team-based visits. The management’s confidence in the team was deemed crucial for fostering trust throughout the whole organization, and vice versa.

*It is very important that you have the support and trust of the management… ​​and that the management values team-based visits and allows time to develop the teamwork… you need to feel listened to.* (Nurse, Group 6)

Continuity within the team was considered important, and management was seen as responsible for scheduling time for team-based visits with flexible teams.

*Team-based visits can be difficult and require continuity.* (Physician, group 1)

However, inaccurate recommendations and paternalistic or educational approaches from team members were described as having resulted in mistrust, as well as confused and insecure children and parents.

*It’s so fragile in a way; a bad experience can damage quite a lot.* (Nurse, Group 6)

Professionals expressed disappointment with managements and MCHUs that failed to ensure that interested and competent professionals were working within CHS. Competing interests between management and professionals were considered as leading to inequalities and inequities within CHS.

### Competence and interprofessional learning

Participants expressed that optimal team-based visits enabled a holistic perspective, providing a comprehensive, high quality, and safe support for children.

*More eyes that see and increased competence…* (Nurse, group 6)

Families’ needs were described as indicative of determining the team composition. The participants regarded the core team in CHS as being a nurse and a physician, extended with other professionals’ competence when needed. However, psychologists were requested more continuously and in a structured manner during the team-based visits.

*We psychologists could, just like physicians,contribute so much… come in early… and talk about things that are strange and scary… that would be my version of the psychologist’s role… clear efforts early and in a preventive approach, even more formalized.* (Psychologist, Group 3)

Fully-fledged team members were considered as having profession-specific knowledge and competence in CHS and team competence. Professionals could learn from each other’s experiences and knowledge, supporting one another in the meetings with the children and the parents. The local management and MCHUs were expected to allocate human resources and enable interprofessional education to ensure that professionals in CHS had the relevant skills and expertise needed for team-based visits. Having a supportive organization also meant prioritizing team-based visits in CHS by scheduling time, preparatory material, education, and documentation adapted for team-based visits in CHS. Organizational benefits described by all professional groups included efficient use of resources and timesaving aspects.

*… we could probably have reduced the number of referrals quite significantly by being able to have more opportunities for team-based visits…* (Psychologist, Group 2).

However, participants expressed frustration, a sense of strain, and stress in team-based visits, when team-members lacked competence, responsibility, or had a negative approach. The consequences of dysfunctional team-based visits, where teams failed to complement each other, resulted in additional work.

*We’ve seen physicians say incorrect things and then it gets crazy. Then I’m going to like change this…and that can affect future visits badly.* (Nurse, Group 6)

Both local and national differences and inequalities regarding support for team-based visits, content, and availability were described as an ineffective use of professional competence.

## Discussion

Well-functioning team-based visits in CHS were highly valued by the professionals in our study, considered as an efficient and comprehensive way to meet the unique needs of children and families. Participants claimed that when team-based visits were delivered correctly, with the right individuals at the right time, they could maintain synergistic effects on all those involved. However, some participants experienced dysfunctional team-based visits with poor quality of care and insufficient family support, both in terms of content and quantity. Without professional team competence, internal and external organizational team-enabling support, team-based visits could have the opposite effect on all parties involved. The FGDs yielded a deeper understanding of the opportunities and difficulties associated with team-based visits in the CHS-context, highlighting a complex interplay between individuals within the teams, the children and families, and the organizational structures. How teams and organizations choose to handle challenges related to team-based visits greatly influence their ability to achieve their goals.

### Child in the center

The present findings indicate that team-based visits, tailored to the family’s needs, are perceived as timesaving for both families and the healthcare organization. Additionally, they promote the child’s perspective and ensure the quality of care. Our results, as well as previous research, found interprofessional collaboration to be suitable for meeting the complex needs of patients [26, 32]. The Swedish NCHP includes universal and targeted team-based visits to meet the unique needs of the child and his or her family [8]. However, a previous study shows that healthcare professionals perceive a greater need for targeted team-based visits than what is currently available [10]. Burström et al. (2017) provide a practical example of targeted team-based visits, involving social workers and CHC-nurses participating in home visits to all newborns in a disadvantaged area in Sweden [21]. According to the Swedish legislation on the Convention on the Rights of the Child, all children have the right to CHS that match their unique needs [1]. The Healthcare Act (2017:30), Chap. 3, § 1 and § 2 in the Swedish law, designates health promotion and prevention as the second most important issue, with a priority for those with the greatest healthcare needs (Swedish Government’s Proposition 1996/97:60). In line with our findings, NCHPs must be flexible and individualized to allow each child to reach their full potential in terms of health and development [1]. According to the three-tier NCHP, the children’s and families’ unique needs should govern the content in the team-based visits and the professionals participating in the team-based visits.

Professionals viewed team-based visits as being interdependent on their own management, as well as other organizations. To meet the unique needs of children and families, team-based visits that are organized around the children and their families are necessary. Our results, together with previous research, indicate the importance of adopting a child centered organizational principle to realize team-based visits in CHS. At a broader level, a prerequisite is that health authorities recognize the importance of teamwork and its associated challenges in the field of CHS, allocating resources and organizing team-based visits according to the needs of the child and his or her family. In our study, co-location around children and families was considered a facilitator for framing the team-based visits. Dierckx (2020) and Nygren et al. (2021) found that a co-location of child and family services (e.g., FCs) in everyday practice was an enabler for continuity and collaborations within the framework of proportionate universalism [10, 33]. However, NCHPs and organizational structures as co-location cannot replace the work within the processes of the team and the individual responsibilities and competence needed for optimal team-based visits.

### Shared responsibilities and common goals

In team-based visits, children are the most important, with professionals there to support them. This study provides new insights into the importance of professionals’ individual competences, clarity in the professionals’ roles, and explicit instructions on how to optimize team-based visits in CHS. According to Reeves et al. (2018) and Nelson et al. (2007), professionals in the team’s composition need to be matched with the team’s purpose to meet each patient’s specific needs [24, 34]. In our study, participants described the nurse’s role in identifying children and families in need of extended support. Engström (2023) indicates that the Safe Environment for Every Kid (SEEK) can empower nurses to identify and assist families in need of targeted interventions [35]. This research, together with previous findings [10], shows the importance of structured universal interventions by individual professionals and teams to deliver targeted team-based visits. Some participants described nurses as team-leaders, while others gave examples of team-based visits, where the team-member with the most appropriate knowledge took the formal leadership role. Reeves et al. (2018) describe the need for shared responsibility [34], and Herchline (2022) emphasizes the importance of flexible leadership within teams [36]. The complexity of health determinants influencing children’s health and development requires different areas of knowledge; thus, the team member with the most expertise or experience in the situation at hand could be the most appropriate professional to guide the team-based visit at that moment. Simultaneously, trusting relationships could be decisive in determining who leads the team-based visits.

### Supportive approach and continuous trustful relations

Our results show that trust between children, families, and team members is a promoting factor for productive team-based visits, but it could also result from team-based visits. Establishing continuous, trustful relationships between nurses and families was considered as basic when building trust within the team and between teams and families. Additionally, maintaining continuity within the team was also perceived as an important factor for optimal team-based visits. Previous research show that organizational structures can encourage continuity and occasions for communication within the team [23, 26, 33]. Internationally, as well as nationally in Sweden, nurses (public health nurses or health visitors) seem to be the most common first contact for CHS [12]. From an organizational design perspective, the development of shared goals for team-based visits in CHS and ensuring that professionals who are interested in CHS are assigned relevant tasks within CHS are crucial aspects for facilitating collaboration. To develop shared goals, team members must engage in communication to get to know each other and build trust. As shown in this research as a whole, and emphasized by other sources, stability within the CHC team’s framing, e.g., norms, values, and goals, is important for the successful implementation and teamwork in CHS [23, 25, 37]. The interplay within the CHC team, as well as between the team and its organization, and the results that follow from the team’s interactions with the child and the family, are always, in different ways, the background for the experiences of trust or mistrust among participants in this context. These experiences, in turn, can be considered as important prerequisites in the treatment process, heavily influencing the development of the family and the child, and bringing the implementation of teamwork to a new level of achievement and consciousness [38]. Adler et al. (2001) consider shared values as a basis for being trustworthy, and confidence as essential in knowledge-intensive healthcare [38]. Overall, in our study, participants thought that children and families placed trust in the teams at the CHS. Team-based visits, embedded within a continuous trustful context, were seen as facilitators of empowerment for children and families. However, there were challenges with some children and families who lacked trust in healthcare professionals and healthcare organizations. Individual team members must have a health-promoting approach that empowers both children and parents. If participating professionals adopt a paternalistic approach, the synergistic effects of the team-based visit can be reversed.

Our findings showed various benefits, such as work-related well-being and a sense of meaningful context, as outcomes of having well-functioning team-based visits. However, additional work and stress were described as possible outcomes from dysfunctional team-based visits. According to Sandberg (2010), *“Teamwork affects team members’ wellbeing and health*,* and the team members’ wellbeing and health affect the teamwork”* (p. 650, rows 41–41) [39]. Wei (2019, 2021) illustrates how a positive and supportive team climate can affect professionals’ adherence to team tasks, knowledge sharing, holistic perspectives, quality of care, and shared problem-solving in complex matters [25, 28]. The interplay between the prerequisites for a well-functioning team-based visit, the structures and processes within the team, and the outcome need to be addressed when implementing and evaluating team-based visits in Swedish CHS. The evaluation of team-based visits should involve real teams, i.e., teams in the true sense of the concept. Negative outcomes resulting from dysfunctional team-based visits could hinder the successful implementation of team-based visits in CHS.

### Competence and interprofessional learning

Our study reveals that trustworthiness is closely associated with the professional’s individual attitude and competence. Participants require profession-specific knowledge, competence in CHC, and team competence for a well-functioning team-based visit. Rydenfäldt et al. (2017) pointed out that for team members to collaborate effectively on the same task (team-based visits), there must be some interdependence in their competences [23]. This reasoning indicates the importance of having the right professional for the specific task. The results indicate a need for comparison between the standards for competence among health professionals in CHS, the perceived competence required within CHS, and the content of specialist education needed for employment in CHS. Inaccurate advice from other team members resulted in families losing trust. However, the lack of knowledge among other team members could be indicative of the need for team-based visits and interprofessional learning within the team. In order to enable interprofessional learning within the team, managers need to organize teams with professionals who are interested and willing to learn. The participants questioned the management’s responsibility for providing interested and competent professionals within the CHS. Team competence and patient-centered care are core competences for healthcare professionals [28, 32]. Knowledge concerning team members’ competence is important to identify individual gaps in knowledge and experience and to monitor interprofessional education [26, 36]. Lack of adequate education or team training could be a barrier for teamwork [25]. In Sweden, there are regional differences in how the CHS is organized. Different management groups could be responsible for providing nurses, physicians, and psychologists. These local management groups may have competing interests. Participants mentioned the importance of MCHU in implementing new methods and ensuring competences through interprofessional education and quality assurance within CHS. Previous research has highlighted the importance of clear leadership [25, 37]. However, a division in leadership at the organizational level could be a hindrance to shared norms, values, and goals at all levels within the organization.

### Methodological considerations

Even though the participants emphasized the positive processes, they also acknowledged problems that could arise. Their overall positive attitude toward team-based visits might be because these participants have chosen to be part of this study and the fact that they contributed to the team’s common competence in their clinical practice. Voluntary participation and participants primarily recruited via MCHUs and professional networks may have skewed the data toward professionals with a positive view of team-based visits or more experience with interprofessional collaboration. Another limitation that emerged was that despite national guidelines, the definition and structure of team-based visits appear to vary regionally, affecting consistency across participant experiences.

To ensure the trustworthiness of the findings, the following criteria must be met: credibility, transferability, dependability, and reflexivity. To establish confidence in that the results (from the perspective of the participants) are true, credible, and believable, all authors discussed the data and the categories during the analysis process. To enhance the quality of the results, researchers with experiences of team and qualitative research, as well as experiences of CHS, were involved in the investigator triangulation. The authors represent different professions and scientific backgrounds, allowing for the possibility of triangulation during the analysis process [40]. Usually, homogeneous groups (e.g., the same professional background) are used in FGDs to promote a comfortable group dynamic. However, we tested a pilot FGD, including nurses, physicians, and psychologists, to evaluate the depth of the discussion. The pilot group worked well. To ensure balance within the groups, we chose profession-specific groups. Thus, the groups were homogeneous regarding the profession, but they had variations regarding individual experiences and working contexts. To enhance credibility, participants with experience in team-based visits were recruited through the MCHUs with knowledge of professionals’ experiences in the healthcare regions. Some participants worked in the same healthcare region as the moderator. It cannot be ruled out that these participants felt a responsibility to participate in the FGDs and thus expressed a particular response. However, we assured the participants in the FGDs that there were no right or wrong answers. Dependability of the research was supported by a clear description of the study context, characteristics of the participants, the data collection, and the steps included in the analysis process. Simultaneously, dependability can be hard to achieve in qualitative studies, since the findings and the results were formed in the meeting between the moderator and the participants in the FGDs. Transferability is also limited by the fact that the study population was restricted to health professionals in the Swedish CHS, and there are differences in CHS practices internationally. However, team-based visits could be practiced in other healthcare contexts, and enabling factors for well-functioning teams could be transferred to these contexts. The reflexivity in this study was obtained through the researchers’ complementary knowledge and experiences. They discussed and reflected critically in the analysis process.

One limitation in this study was the possibility for interpersonal interaction. The FGDs were conducted during the Covid-19 pandemic, during which time it was difficult to hold physical group meetings. However, the fact that the FGDs were digital made it possible for participants from different healthcare regions in Sweden to meet. Even if the digital format is not optimal for interaction between participants, it still worked well because the participants were used to meeting digitally. However, the recruitment of participants was impacted due to the Covid-19 pandemic.

Finally, the study exclusively focused on professionals’ perspectives, omitting the views of children and parents, who are central to evaluating the effectiveness, equity, and responsiveness of team-based visits. The study provides cross-sectional insight into professional’s experiences and perceptions. There is a need for knowledge about long-term outcomes or effects of team-based visits on actual child health indicators.

## Conclusions

This study provides new insights into the short- and long-term organizational benefits of optimal teams within the CHS, particularly as it works with team-based visits in a Swedish context. The uniqueness of this study is that it is the first to explore team-based visits within the CHS context. Our findings show great opportunities for promoting children’s health and preventing illness through a holistic approach in team-based visits. Professionals described positive attitudes and adequate competences as essential to meet the unique needs of children and families through team-based visits. Trustful relationships, communication, shared goals, norms, and values and shared responsibly were deemed important processes. Additionally, a child- and family-centered approach was considered essential. The content and roles within the team-based visits, as well as continuity, were seen as essential structures. From an organizational perspective, structures, resources, and support were required at the national, regional, and local levels. In the case of several stakeholders involved in the local management, shared goals and responsibilities are needed. With the best interests of the child at the center, organizational support and the provision of competence and resources are necessary for realizing optimal team-based visits within the CHS context. In line with children’s rights to have the highest attainable standard of health and access to CHS, the organization of team-based visits should be prioritized and approached from a child- and family-centered perspective. However, without enabling conditions, structures, and processes within the team or the context where the team operates, team-based visits could have the opposite effect for all those involved.

## Supplementary Information

Supplementary Material 1.

## Data Availability

The data will be available from the corresponding author upon reasonable request.

## Abbreviations

CHC: Child healthcare center
CHS: Child healthcare service
FC: Family center
FGD: Focus group discussion
MCHU: Main child healthcare units
NCHP: National child healthcare program
